# The relative validity of the updated Menzies Remote Short-Item Dietary Assessment Tool (MRSDAT) for use with remote Aboriginal and Torres Strait Islander children and adults

**DOI:** 10.1186/s12889-025-23233-x

**Published:** 2025-05-29

**Authors:** Emma Tonkin, Ellie Chan, Caroline Deen, Bronwyn Fredericks, Morag Dhurrkay, Hasthi U. Dissayanake, J. Dhurrkay, George Gurruwiwi, Beverley-Ann Biggs, Julie Brimblecombe

**Affiliations:** 1 Department of Nutrition, Dietetics & Food, Monash University, Notting Hill, VIC 3168 Australia; 2 School of Public Health, The University of Queensland, Herston, QLD 4006 Australia; 3 Central Australian Aboriginal Congress, Alice Springs, NT 0870 Australia; 4 Apunipima Cape York Health Council, Bungalow, QLD 4870 Australia; 5 Office of the Deputy Vice-Chancellor, The University of Queensland, Herston, QLD 4006 Australia; 6 Australian Research Council Centre of Excellence for Indigenous Futures, The University of Queensland, St Lucia, QLD 4072 Australia; 7 Department of Infectious Diseases, The Doherty Institute, University of Melbourne, 3000, and Victorian Infectious Diseases, Royal Melbourne Hospital, Melbourne, 3050 Australia; 8 Menzies School of Health Research, Casuarina, NT 0810 Australia

**Keywords:** Diet, Questionnaire, Indigenous, First Nations, Public health, Food, Australia, Dietary assessment

## Abstract

**Background:**

This study aimed to determine the relative validity of dietary quality index scores estimated using the updated version of the Menzies Remote Short-item Dietary Assessment Tool (MRSDAT), a food frequency questionnaire developed for use with remote Indigenous communities in Australia.

**Methods:**

This validation study compares scores derived from the updated MRSDAT with those derived from 24-h recalls. Participants from nine remote communities across three discrete regions of Australia (Central Australia, North Northern Territory and Cape York) participated in June–September 2021, or May–July 2022. Participants were Indigenous children aged six months to five years, pregnant and/or breastfeeding Indigenous women, and overweight adult Indigenous males and females. The agreement between methods was explored using Lin’s concordance correlation coefficient (CCC), Bland–Altman plots, weighted Cohen’s kappa, paired samples t-tests and Wilcoxon signed-rank tests. Primary comparisons were completed for the whole sample, and primary and secondary comparisons for only participants with ≥ 2 × 24 h recalls.

**Results:**

Ninety-nine participants were recruited. The MRSDAT estimated dietary quality index scores 0.31/100 points higher than 24-h recall estimates for participants with ≥ 2 × 24-h recalls, and 0.18/100 points higher for all participants, with consistent results for adults and children (CCC = Rc 0.58, 95% CI 0.39, 0.72).

**Conclusions:**

Updates to the MRSDAT have resulted in dietary quality index scores highly comparable to those derived from 24-h recalls. The MRSDAT has the practical advantages of speed and convenience for research purposes, and the built-in instant dietary feedback feature has application to service delivery settings given the robust results demonstrated in this validation study.

## Introduction

### Background

Accurate dietary data at the individual level is required for a number of purposes: to plan and evaluate health and nutrition policies, to evaluate community-led programs, and for nutritional status and diet quality monitoring to determine progress towards reducing inequities in the social determinants of health which underpin dietary choices [1, 2]. Dietary assessment is not without methodological complexity however, and all forms of dietary assessment include inherent bias, most being vulnerable to issues of self-report data such as underreporting and social-desirability bias [2, 3]. New technologies which assist in removing these biases, such as the use of photographs to document intake [4], are currently too resource intensive for participants, researchers, and practitioners to be commonly used in research and practice settings [2, 5, 6]. Therefore, increasing attention is being paid to the development of dietary metrics derived using food frequency questionnaires (FFQs) which can be conducted conveniently with large populations to summarise aspects of the diet such as adequacy, quality and diversity, with a number even validated against health and non-communicable disease outcomes [7].

While some dietary scores and metrics have been developed with the intention to be used globally [7], differences in dietary patterns across populations make the use of one standardised tool for all populations inappropriate. This is especially so for Aboriginal and Torres Strait Islander populations in Australia [1]. Aboriginal and Torres Strait Islander peoples engage with both the traditional and Western food systems [8–12]. This leads to their food environments being described in a range of ways, including that Indigenous peoples are living in ‘two cultural worlds’ [13] or ‘both-ways’ [14]. Others understand it as all part of living in the contemporary world, where people have greater access to a range of foods, and can make choices based on availability, preferences, lifestyles, natural disasters, and recently lockdowns, along with cultures [15–17]. Traditional foods consumed include diverse animal (mammal, bird, reptile, insect and marine) and plant species (roots, legumes, seeds, nuts, fruits, nectars, flowers and gums) which are highly nutrient dense [12, 18, 19]. While one consequence of colonisation has been the disruption of access to traditional foods for Aboriginal and Torres Strait Islander peoples, these foods remain an important part of the diet of many Aboriginal and Torres Strait Islander groups in Australia [20], particularly those living in remote communities [13, 21] where there is greater availability, and where people may be living on or close to their own lands and waters, or are able to access the land and waters of other Aboriginal and Torres Strait Islander peoples [9, 11, 18, 19, 22–24]. Many of these foods are not typically captured in dietary assessment tools designed from the perspective of the Western food system.

The Menzies Remote Short-Item Dietary Assessment Tool (MRSDAT) is a 32-item FFQ originally developed for the mothers and children of the Pregnancy and Adverse Neonatal Diabetes Outcomes in Remote Australia (PANDORA) longitudinal birth cohort study [25]. The MRSDAT was developed with the intention of being a tool designed to be appropriate for use with remote Aboriginal and Torres Strait Islander communities, and its development has been previously described [26]. A Dietary Guideline Index (DGI) score was later derived from the MRSDAT [27] based on a diet quality scoring system originally developed for both adults (DGI) and children (DGI-CA) in the general Australian population [28–31]. The DGI/DGI-CA scores are estimates of diet quality, and provide a numerical comparison of the measured diets with the intake and serving amounts as recommended within the Australian Dietary Guideline Foundation Diets (ADGs) [32]. In addition, other aspects of diet quality reflected in the dietary guidelines, such as choosing plain water, unsweetened dairy, wholegrain cereals and unprocessed meats are captured in both questions and scoring. The MRSDAT derived DGI scores were validated for use with children aged 6–36 months in one remote Aboriginal community, with performance consistent with other short-item food frequency dietary assessment tools [27]. Recently, the MRSDAT has been further developed by a team of Aboriginal and non-Indigenous researchers to address issues identified in the previous validation, and to extend its use across all age groups and genders included in the ADGs. Additionally, it has been programmed into an online platform, with the DGI/DGI-CA scoring system automated, enabling instant feedback regarding food group intakes compared to age group/life-stage recommendations, and DGI/DGI-CA scores for participants and researchers. This study therefore explores the dietary quality scoring performance of the updated and extended, online version of the MRSDAT tool.

### Aim

This study aimed to determine the relative validity of the DGI-CA score for Indigenous children aged six months to five years, and the DGI score for pregnant and/or breastfeeding Indigenous women, and overweight adult Indigenous males and females, derived using the updated version of the MRSDAT.

## Methods

### Design

This study compares DGI and DGI-CA scores derived from the updated MRSDAT with those derived from 24-h recalls. While weighed food records are known to produce less bias than 24-h recalls [2], the participant burden was thought to be unacceptable for the participants of this study. In all populations 24-h recalls are vulnerable to underreporting of intake due to forgotten foods and portion underestimation, however they are thought to be the most appropriate reference measure in studies where there is a divergence in language and literacy between participants and researchers [2, 33, 34]. Additionally, they are the most common dietary assessment method used for assessing individual-level diet in Aboriginal and Torres Strait Islander populations aside from FFQs [1], and are currently used for national nutrition monitoring [3], making a comparison of estimates from the MRSDAT and 24-h recalls meaningful.

These data are drawn from data collected as part of two separate trials conducted in remote Aboriginal and Torres Strait Islander communities. The Remote Food Security study [35] tested the effect of price discounts on a range of healthy foods for eligible women and parents/primary carers of eligible children. The pre–post intervention study of the Hope for Health program evaluated an on-Country nutrition and lifestyle program for improvement in body weight and cardiometabolic markers. All aspects of the dietary intake assessment component of both trials were overseen by the same team of experienced researcher dietitians. Therefore, consistency was achieved in the design, methods, tools, implementation, and training provided to data collection teams.

#### Ethics

The studies were approved by the Research Governance Committee of Apunipima Cape York Health Council, The Central Australian Aboriginal Congress Board, Miwatj Health, the East Arnhem Regional Council, Central Australian Human Research Ethics Committee (CA-20–3701), The University of Queensland Human Research Ethics Committee B (2020000636) and The Human Research Ethics Committee of The Northern Territory Health and Menzies School of Health Research (2021–4166). Written informed consent was provided by each participant, or their caregiver, after study procedures had been explained.

### Participants

The trials were conducted across nine remote communities in total: four in Central Australia in the Northern Territory, four in Cape York in Queensland, and one in the North of the Northern Territory. All the communities are considered very remote [36]. The communities span a diversity of geographical regions and climate, including four arid, desert communities and five tropical, coastal communities.

In the Remote Food Security study, dietary data were collected for Aboriginal and/or Torres Strait Islander pregnant and breastfeeding women, and children aged 6 months to five years. Local services provided lists of community members eligible for the study, with community researchers assisting to locate potential participants to invite their participation. Recruitment was conducted at participants’ homes, at the community health clinics or family centres. After consenting to participate in the trial, and completing all components of the trial data collection, participants were asked if they would like to additionally participate through providing 24-h recall data. The procedure was outlined, and separate consent was provided if the participant agreed. Participants were reimbursed to the value of $20 for each 24-h recall completed.

In the Hope for Health program trial, dietary data were collected from adults aged 18–65 years with body mass index ≥ 25 kg/m2 (or waist circumference > 94 cm for males and > 80 cm for females), without unstable or insulin dependent diabetes, chronic renal failure, cardiac failure, cirrhosis or pregnancy. Recruitment involved research teams (including local researchers) visiting each house within the community to provide trial information, ascertain interest, and screen participants for trial eligibility. Those eligible and interested attended a clinic where trial procedures were explained in both English and local language, consent was provided, and data collected.

### Data collection

In both trials dietary assessment was planned to be completed over two or three sessions, depending on the needs of the participant. As the new method, and the primary dietary assessment method for both trials, the MRSDAT was always administered first as part of the full baseline data collection for each trial. If consent was provided, this was followed by the first 24-h recall [33]. Occasionally the first 24-h recall was completed on another day of the week due to participant fatigue from completing the full trial baseline data collection prior to the MRSDAT. Given the MRSDAT aims to capture usual intake over a fortnightly period, the second 24-h recalls were then conducted over a 2–3 week period, ideally aiming to capture a pay week day, non-pay week day and weekend day given the variability in intakes during the pay cycle documented in this population [22].

All dietary assessments were completed by the same three research teams (one in each region). Each team consisted of a senior nutrition researcher (JB) or project manager (EC, CD), who were all trained dietitians experienced in working with remote Aboriginal and Torres Strait Islander populations, local researchers, and additional research staff. The senior nutrition researcher or project managers either completed or oversaw all sessions, supported by local researchers and other staff where necessary. Local researchers were integral in supporting participants and translating questions and responses into local language when needed.

#### MRSDAT

Remote Food Security study data were collected in June–September 2021, and Hope for Health program trial data collected May–July 2022, at a location the participant chose. All research team members were trained in the use of the MRSDAT by the same experienced research dietitians (ET, JB). The MRSDAT was completed via Qualtrics (Qualtrics International, USA), either online or using the offline app, on iPads/tablets or phones, and was always facilitated by researchers [2]. Researchers most commonly read questions and entered participants’ verbal responses into Qualtrics, but occasionally participants held the device and entered their own responses, asking questions of researchers when clarification was required. Illustrative food serving images are included in the Qualtrics version of the MRSDAT to assist with respondent estimation of serving size. Images include food type prompts (e.g. different types of vegetables and sugar sweetened beverages [SSBs]) and serving size prompts (e.g. an image of a hand with the palm circled captioned ‘one serve of meat’). Researchers were guided to suggest to participants a reference period of the preceding fortnight when considering consumption frequency to capture ‘usual’ short-term intake. Participants in the Remote Food Security study were offered the opportunity to receive immediate dietary feedback using the automated scoring function at follow up data collection.

#### Updates to the previously validated MRSDAT

The changes to the previously described [26] and validated [27] MRSDAT are as follows. Separate versions have been developed for young children (child version), and older children and adults (self-complete version). Serve size response options have been adapted to be appropriate for the age groups in each version; for example, the response options for meat have been extended to include smaller serves for the child version (e.g. ‘0.5 serves or less’, ‘0.5–1 serve’ and ‘more than 1 serve’), and larger serves for the self-complete version (up to ‘5 serves or more’). Additional questions to capture frequency and volume of dairy intake, as well as plain water, chips/crackers and processed meat consumption were included. Red and white meat questions were collapsed into the single question ‘How many serves of fresh or frozen land animals and/or birds does your child usually eat per day (includes kangaroo, beef, lamb, chicken, turkey, pork, duck, magpie goose, *does not include processed meats*)?’. The previous open-ended response option for SSB consumption was replaced with a multiple-choice response. Finally, five questions that did not contribute to the DGI score were removed.

#### 24-h recalls

A standardised three-pass 24-h recall procedure was used for all recall sessions [37, 38]. The participant or child’s caregiver was asked to recall everything consumed in the previous 24 h, with quantities estimated using household measures provided by the researchers when needed (metric cups and spoons). Participants were free to choose whether they reported on the exact preceding 24-h period, or midnight-to-midnight the preceding day. Researchers noted responses by hand on a standardised hardcopy recording sheet. For 24-h recalls of child diets, the caregiver was asked if anyone else had spent time caring for the child during the relevant 24-h period, including other children, and if available, these people were followed up and details of intake recorded. Data collectors had all familiarised themselves with the food products available in the community store and take-aways, and therefore were able to prompt with detail about forgotten foods, portion sizes and brands. A standard set of cup and spoon measures were used to assist with portion size. Hardcopy data were entered into a custom made Access database (Microsoft, USA) built using the 2011–13 AUSNUT food composition database [39], and the Australian Health Survey discretionary food list and food and supplement classification data cubes [40]. All data were entered by the project managers for two regions (EC, CD), and by additional research staff for the third. The complete database for each region was cleaned and cross-checked against all hard copies by the same third researcher to ensure consistency in data entry (ET).

### Analysis

#### Conversion of both datasets to food groups and DGI/DGI-CA scores

Intake data from the 24-h recalls were converted to, and the online MRSDAT automatically calculates, daily food group servings using reference servings from the ADGs [32]. Daily intake data from multiple 24-h recalls were then averaged to calculate average daily food group servings for each participant, which were checked for plausibility. These data were then used to calculate diet quality scores derived from the averaged 24-h recalls using statistical software, to compare with those automatically derived from the MRSDAT.

The DGI scoring method used was based on the original DGI-CA for children aged 4–11 years [28, 30], with modifications as outlined in [27]. Therefore, the DGI/DGI-CA score used here reflects key elements of the Australian dietary guidelines, comprising eleven ‘indicators’; 10 points are allocated for each of the five food groups (fruit, vegetables, meat and alternatives, dairy and breads and cereals [5 points each for intake and wholegrain quality]), 10 points each for two additional recommendations (dietary variety and consumption of healthy fats), five points each for plain water consumption and no intake of SSBs, and finally 20 points for no intake of discretionary foods. For example, if the recommended daily vegetable intake for a participant is 4 serves, and the participant consumed 2 serves (half the recommended intake), they scored 5 out of a possible 10 points for the vegetable indicator (half the possible points). Total DGI and DGI-CA scores are calculated by summing all indicator scores, with a maximum possible score of 90 for children under two years due to the exclusion of the dairy indicator (see [27]), and 100 for all participants > 2 years. DGI-CA scores for participants < 2 years were here multiplied by 10/9 to create a score out of 100 for comparability with the other age groups.

#### Statistical analyses

Food group intakes, indicator scores, and DGI scores derived from the MRSDAT and 24-h recalls are presented. The primary method for exploring relative validity was Lin’s Concordance Correlation Coefficient (Lin’s CCC) [41, 42]. Unlike other methods of correlation, Lin’s CCC accounts for both the linear relationship between, and the slope of the line relating, the sets of DGI/DGI-CA estimates (that is, the estimated diet quality scores) from each method, and requires a minimum of 10 pairs of data to be robust [42]. Bland–Altman plots, including mean difference, limits of agreement (LOA) and proportional bias assessed via linear regression were also used to graphically explore agreement, while weighted Cohen’s kappa was used to compare how each method ranked participants into tertiles [33, 43]. Secondary comparisons of food group intakes and individual indicator scores used paired samples *t*-tests or Wilcoxon signed-rank tests. Primary comparisons were completed for the whole sample, and primary and secondary comparisons for only participants with ≥ 2 × 24 h recalls (pairs only) given the documented need for multiple 24-h recalls to estimate usual intake [2]. Sub-analysis included splitting the whole sample and pairs only into adult and child groupings. To account for multiple comparisons *p*-values were adjusted with Bonferroni's correction [44, 45], with significance set at *p* < 0.05. IBM SPSS Statistics version 29 (IBM Corp, USA) was used for statistical analyses.

## Results

Participant demographics are presented in Table 1. A complete MRSDAT and at least one 24-h recall were available for 99 participants across 13 ADG age categories. Sixty-two completed two 24-h recalls. The median time between recalls was 6 (IQR 3, 12) days. The mean completion time for MRSDATs was 15.7 (SD 6.8) minutes, and 136 (77%) of the 177 Remote Food Security participants offered the opportunity to receive the immediate auto-scored dietary feedback accepted. Although 24-h recalls were not formally timed, data collectors estimate they ranged from 10–40 min to complete, typically taking less time for young children with less intake. The 24-h recall session was reported by the participant to reflect their/the child’s usual intake for 118 (73%) recalls overall (Table 1). For children the reasons for eating less than usual included special occasions, child not hungry or refusing to eat, and being with another carer; reasons for eating more than usual were special occasions or unusually hungry. For adults, reasons for eating less than usual were illness, dental problems, not being hungry, fasting, having no money for food or deliberately skipping meals to lose weight; while eating more than usual was due to being unusually hungry, it being pay day, going hunting, or being provided food by a service. One participant who completed only a single recall, had almost no food intake due to illness, and reported this was much less than usual, was excluded from further analysis.

**Table 1:** Participant characteristics and reports of usual intake

	**Single 24-h recall, *n* (%)**	** ≥ 2 × 24-h recalls, *n* (%)**
Region		
North Northern Territory	45 (45)	20 (32)
Central Australia	18 (18)	16 (26)
Cape York	36 (36)	26 (42)
Age		
Child 6—< 24 months	7 (7)	6 (10)
Child 2—< 4 years	13 (13)	10 (16)
Child 4 – < 6 years	11 (11)	10 (16)
Pregnant and/or breastfeeding female	23 (23)	16 (26)
Male 19–50	12 (12)	4 (7)
Female 19–50	23 (23)	10 (16)
Male 51–70	3 (3)	2 (3)
Female 51–70	7 (7)	4 (7)
Reports of usual intake		
Usual intake	69 (70)	49 (79)
Less than usual	17 (17)	10 (16)
More than usual	11 (11)	3 (5)
Don’t know	2 (2)	-

### Comparison of daily food group intakes

Table 2 provides a comparison of the daily food group intakes estimated by the two methods, including comparison by subgroup. Analysis of both single and paired samples show both methods estimated food group intakes similarly. Although there were no significant differences between estimates overall, SSB and discretionary intakes were estimated to be slightly higher by the MRSDAT, and sub-analyses showed this to be primarily driven by divergence in the estimates for children, although these were not significant. For children the MRSDAT had a 0.65 serve lower estimate of meat (*p* = 0.03), and while this was not the case in adults, adults had a 0.66 serve higher estimate of fruit intake with the MRSDAT (*p* = 0.03).

**Table 2:** Comparison of the median daily food group intakes derived from the MRSDAT and the 24-h recalls

Food group	MRSDAT (all), median serves/day (IQR)	Average of 24-h recalls (all), median serves/day (IQR)	MRSDAT (pairs only)^a^, median serves/day (IQR)	Average of 24-h recalls (pairs only), median serves/day (IQR)	Z-statistic, *p*-value for comparison of estimates from pairs only
**All participants**					
	***n* = 98**		***n* = 62**		
Vegetables	1.00 (0.55, 2.14)	1.47 (0.64, 3.18)	1.21 (0.51, 2.32)	1.47 (0.73, 2.93)	−0.96, *p* = 1.00
Fruit	0.86 (0.28, 2.00)	0.43 (0.00, 1.04)	1.00 (0.43, 2.00)	1.00 (0.13, 1.42)	−2.77, *p* = 0.08
Breads and cereals	5.00 (3.00, 5.50)	5.51 (3.16, 7.61)	5.00 (3.00, 7.00)	5.66 (3.16, 7.79)	−2.68, *p* = 0.10
Meat	2.47 (1.63, 3.64)	2.90 (1.84, 4.11)	2.10 (1.55, 3.58)	2.75 (1.79, 3.59)	−1.99, *p* = 0.66
Dairy^b^	1.47 (1.00, 2.43)	1.08 (0.47, 2.06)	1.86 (1.04, 2.75)	1.21 (0.70, 2.33)	−2.30, *p* = 0.29
Sugar sweetened beverages	0.43 (0.14, 1.56)	0.19 (0.00, 0.74)	0.43 (0.14, 1.79)	0.24 (0.00, 0.83)	−2.77, *p* = 0.08
Discretionary foods	4.00 (2.5, 6.00)	2.54 (1.15, 4.38)	3.75 (2.44, 5.13)	2.54 (1.41, 4.12)	−2.76, *p* = 0.08
**Children**					
	***n* = 31**		***n* = 26**		
Vegetables	1.14 (0.50, 2.29)	1.80 (0.87, 2.91)	1.14 (0.51, 2.33)	2.09 (0.90, 3.00)	−1.03, *p* = 1.00
Fruit	1.29 (0.43, 3.00)	1.06 (0.56, 1.62)	1.15 (0.43, 2.25)	1.18 (1.00, 1.69)	−0.77, *p* = 1.00
Breads and cereals	3.00 (3.00, 5.00)	4.03 (2.84, 5.93)	3.00 (3.00, 5.00)	4.15 (2.81, 5.94)	−1.18, *p* = 1.00
Meat	1.43 (1.03, 1.96)	2.03 (1.31, 2.57)	**1.43 (1.03, 1.96)**	**2.08 (1.44, 2.80)**	**−3.16, *p* = 0.03**
Dairy^b^	2.14 (1.43, 3.00)	1.63 (0.22, 2.70)	2.14 (1.43, 2.97)	1.63 (0.37, 2.78)	−2.46, *p* = 0.20
Sugar sweetened beverages	0.43 (0.21, 0.43)	0.14 (0.00, 0.47)	0.43 (0.19, 0.43)	0.12 (0.00, 0.37)	−2.57, *p* = 0.14
Discretionary foods	3.25 (1.75, 4.25)	2.00 (0.83, 4.03)	3.38 (2.13, 4.25)	1.98 (0.80, 4.13)	−1.94, *p* = 0.73
**Adults**					
	***n* = 67**		***n* = 36**		
Vegetables	1.00 (0.56, 2.14)	1.42 (0.44, 3.32)	1.28 (0.54, 2.39)	1.41 (0.67, 2.42)	−0.47, *p* = 1.00
Fruit	0.70 (0.28, 1.29)	0.12 (0.00, 0.96)	**0.86 (0.28, 2.00)**	**0.20 (0.00, 1.00)**	**−3.10, *p* = 0.03**
Breads and cereals	5.00 (5.00, 7.00)	6.35 (4.25, 8.27)	5.00 (3.00, 7.00)	6.47 (4.84, 9.18)	−2.50, *p* = 0.17
Meat	3.15 (2.35, 4.00)	3.49 (2.58, 4.41)	3.36 (2.21, 4.04)	3.19 (2.55, 4.10)	−0.38, *p* = 1.00
Dairy	1.43 (0.86, 2.14)	0.98 (0.46, 1.89)	1.58 (0.90, 2.43)	1.12 (0.71, 1.96)	−1.01, *p* = 1.00
Sugar sweetened beverages	0.43 (0.14, 2.00)	0.31 (0.00, 0.95)	0.43 (0.14, 2.00)	0.53 (0.00, 1.26)	−1.99, *p* = 0.64

^a^Food group intakes derived for only those participants with at least 2 × 24 h recalls (a pair)

^b^excludes 7 participants (1 single, 6 pairs) < 2 years

### Comparison of DGI-CA and DGI indicator scores

#### Overall DGI-CA/DGI scores

**Fig. 1:**
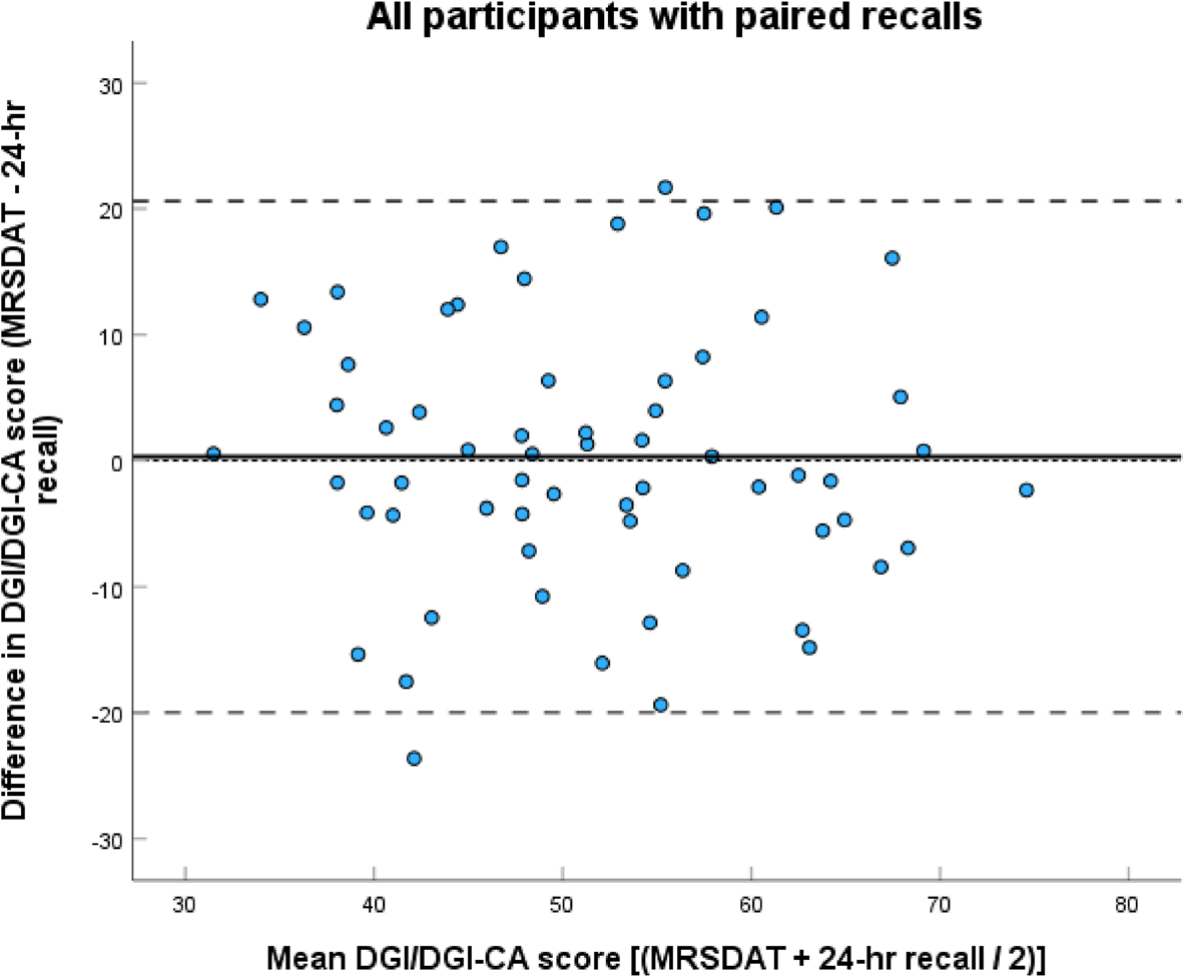
Bland–Altman plot showing agreement between DGI/DGI-CA scores, estimated from the MRSDAT and paired 24-h recalls (*n* = 62) (DGI-CA for < 2 years adjusted to be/100). Mean difference (bias) is represented by the solid line, while the broken lines represent the upper and lower limits of agreement, and the smallest broken line zero

**Fig. 2:**
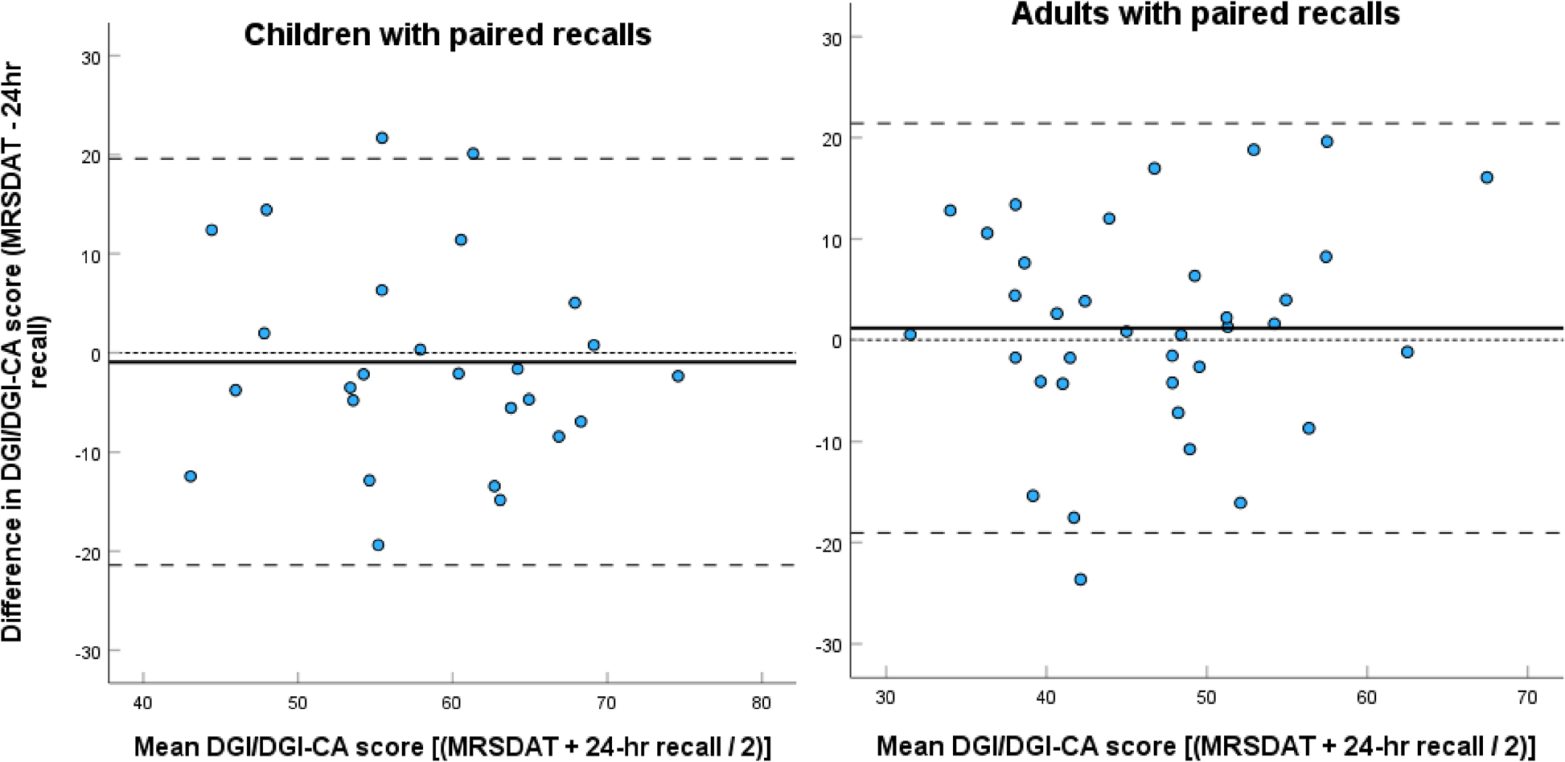
Bland–Altman plots showing agreement between DGI/DGI-CA scores, estimated from the MRSDAT and paired 24-h recalls for children (*n* = 26) and adults (*n* = 36) (DGI-CA for < 2 years adjusted to be/100). Mean difference (bias) is represented by the solid line, while the broken lines represent the upper and lower limits of agreement, and the smallest broken line zero

The Lin’s CCC for the overall DGI-CA/DGI scores derived from the two methods, for participants with ≥ 2 × 24-h recalls was 0.58 (95% CI 0.39, 0.72, *n* = 62), which would be considered to represent a moderate, and almost substantial, agreement. When participants with single recalls were included the Lin’s CCC was 0.54 (95% CI 0.38, 0.66, *n* = 98). Agreement was similar for children (Rc 0.43, 95% CI 0.06, 0.69, *n* = 26) and adults (Rc 0.43, 95% CI 0.12, 0.66, *n* = 36). This was reflected in very high agreement in overall mean DGI scores, with the MRSDAT estimating higher by only 0.31/100 points for participants with ≥ 2 × 24-h recalls, and 0.18/100 points when all participants were included (Table 3). The Bland–Altman graphs presenting data from participants with ≥ 2 × 24-h recalls in Figs. 1 and 2 graphically demonstrate this, and suggest moderate LOA. Regressions of the Bland–Altman data showed no proportional bias for the overall sample (slope −0.04, *p* = 0.78), children (slope −0.16, *p* = 0.54), or adults (slope −0.18, *p* = 0.41), suggesting the MRSDAT and 24-h recall estimates differ to a similar degree above and below the mean, and regardless of score.

**Table 3:** Comparison of individual indicator scores derived from the MRSDAT and the 24-h recalls

Indicator (/possible score)	MRSDAT (all), mean SD, median (IQR)	Average of 24-h recalls (all), mean SD, median (IQR)	MRSDAT (pairs only)^a^, mean SD, median (IQR)	Average of 24-h recalls (pairs only), mean SD, median (IQR)	t-Test or Wilcoxon signed-rank result^#^, (pairs only)
**All participants**					
	***n* = 98**		***n* = 62**		
**Total DGI-CA-DGI score (/100)**	49.68 (11.72)	49.50 (11.30)	51.66 (11.13)	51.35 (11.46)	0.31, *p* = 1.00
**Food group indicators**					
Vegetable (/10)	2.30 (1.14, 4.90)	3.04 (1.39, 6.86)	2.85 (1.33, 5.70)	2.91 (1.49, 6.78)	−0.75, *p* = 1.00
Fruit (/10)	4.30 (1.40, 10)	3.04 (0, 7.24)	6.45 (2.15, 10)	5.03 (0.69, 10)	−1.90, *p* = 0.81
Breads and Cereals (/5)	4.17 (2.78, 5.00)	4.53 (3.44, 5.00)	3.82 (2.78, 5.00)	4.46 (3.54, 5.00)	−1.91, *p* = 0.80
Meat (/10)	10 (9.30, 10)	10 (9.86, 10)	10 (9.47, 10)	10 (9.90, 10)	−0.34, *p* = 1.00
Dairy^b^ (/10)	6.87 (4.00, 10)	4.31 (1.54, 9.06)	8.34 (4.56, 10)	5.17 (2.75, 10)	−2.42, *p* = 0.22
SSB (/5)	2.85 (0, 4.30)	4.07 (1.31, 5.00)	2.85 (0, 4.30)	3.79 (0.69, 5.00)	−2.28, *p* = 0.32
Discretionary foods (/20)	0 (0, 0)	0 (0, 7.85)	0 (0, 0)	0 (0, 3.61)	−2.83, *p* = 0.07
**Other indicators**					
Dietary variety (/10)	6.47 (1.53)	6.08 (1.82)	6.72 (1.54)	6.59 (1.82)	0.13, *p* = 1.00
Healthy fats (/10)	4.91 (2.14)	3.10 (1.40)	**5.02 (1.90)**	**3.21 (1.34)**	**1.81, *p* = 0.01**
Whole grain quality (/5)	2.50 (0, 2.50)	0 (0, 5.00)	2.50 (0, 2.50)	0 (0, 5.00)	−1.31, *p* = 1.00
Plain water (/5)					
Yes	*n* = 96 (98%)	*n* = 96 (98%)	*n* = 61 (98%)	*n* = 62 (100%)	
No	*n* = 2 (2%)	*n* = 2 (2%)	*n* = 1 (2%)	*n* = 0	
**Children**					
	***n* = 31**		***n* = 26**		
**Total DGI-CA-DGI score (/100)**	56.87 (11.03)	57.19 (10.44)	57.87 (9.23)	58.81 (10.34)	0.94, *p* = 1.00
**Food group indicators**					
Vegetable (/10)	4.56 (2.22, 8.60)	6.06 (2.33, 9.95)	5.16 (1.99, 8.96)	6.35 (2.26, 9.96)	−0.82, *p* = 1.00
Fruit (/10)	10 (4.30, 10)	10 (6.67, 10)	10 (6.08, 10)	10 (7.03, 10)	−1.22, *p* = 1.00
Breads and Cereals (/5)	3.75 (3.75, 5.00)	5.00 (3.56, 5.00)	3.75 (3.75, 5.00)	4.96 (3.53, 5.00)	−0.68, *p* = 1.00
Meat (/10)	10 (10, 10)	10 (10, 10)	10 (10, 10)	10 (10, 10)	−0.68, *p* = 1.00
Dairy^b^ (/10)	10 (7.75, 10)	9.53 (1.48, 10)	10 (7.75, 10)	9.53 (2.19, 10)	−2.28, *p* = 0.32
SSB (/5)	2.85 (2.85, 3.95)	4.30 (2.64, 5.00)	**2.85 (2.76, 3.95)**	**4.38 (3.13, 5.00)**	**−2.95, *p* = 0.04**
Discretionary foods (/20)	0 (0, 0)	0 (0, 1.28)	0 (0, 0)	0 (0, 1.84)	−1.84, *p* = 0.92
**Other indicators**					
Dietary variety (/10)	7.24 (1.63)	7.68 (1.56)	7.29 (1.59)	7.96 (1.42)	0.67, *p* = 0.81
Healthy fats (/10)	5.38 (2.41)	2.89 (1.35)	**5.45 (2.16)**	**3.03 (1.38)**	**2.42, *p* = 0.01**
Whole grain quality (/5)	2.50 (0, 2.50)	0 (0, 5.00)	2.50 (0, 2.50)	0 (0, 5.00)	−0.18, *p* = 1.00
Plain water (/5)					
Yes	*n* = 31 (100%)	*n* = 31 (100%)	*n* = 26 (100%)	*n* = 26 (100%)	
No	*n* = 0	*n* = 0	*n* = 0	*n* = 0	
**Adults**					
	***n* = 67**		***n* = 36**		
**Total DGI-CA-DGI score (/100)**	46.35 (10.54)	45.94 (9.88)	47.17 (10.27)	45.96 (9.02)	1.21, *p* = 1.00
**Food group indicators**					
Vegetable (/10)	1.72 (0.85, 3.00)	2.32 (0.74, 6.28)	2.13 (0.92, 4.14)	1.96 (1.28, 3.83)	−0.38, *p* = 1.00
Fruit (/10)	3.50 (1.40, 6.45)	0.59 (0, 4.80)	**4.30 (1.4, 10)**	**1.01 (0.02, 5.00)**	**−3.05, *p* = 0.03**
Breads and Cereals (/5)	4.17 (2.78, 5.00)	4.44 (3.18, 5.00)	3.89 (2.58, 5.00)	4.39 (3.53, 5.00)	−1.76, *p* = 1.00
Meat (/10)	10 (9.30, 10)	10 (9.56, 10)	10 (8.75, 10)	10 (9.57, 10)	−0.16, *p* = 1.00
Dairy (/10)	5.16 (3.44, 8.56)	3.85 (1.54, 7.41)	6.30 (3.00, 9.57)	4.40 (2.75, 7.54)	−1.33, *p* = 1.00
SSB (/5)	2.85 (0, 4.30)	3.45 (0.27, 5.00)	2.85 (0, 4.30)	2.35 (0, 5)	−0.76, *p* = 1.00
Discretionary foods (/20)	0 (0, 0)	0 (0, 9.33)	0 (0, 0)	0 (0, 4.69)	−2.25, *p* = 0.35
**Other indicators**					
Dietary variety (/10)	6.12 (1.35)	5.33 (1.40)	6.31 (1.38)	5.60 (1.39)	0.71, *p* = 0.35
Healthy fats (/10)	4.69 (1.98)	3.20 (1.42)	**4.72 (1.66)**	**3.35 (1.32)**	**1.37, *p* = 0.01**
Whole grain quality (/5)	2.50 (0, 2.50)	0 (0, 5.00)	2.50 (0, 2.50)	2.50 (0, 5.00)	−0.18, *p* = 1.00
Plain water (/5)					
Yes	*n* = 65 (97%)	*n* = 65 (97%)	*n* = 35 (97%)	*n* = 36 (100%)	
No	*n* = 2 (3%)	*n* = 2 (3%)	*n* = 1 (3%)	*n* = 0	

^a^Food group intakes derived for only those participants with at least 2 × 24 h recalls (a pair)

^b^excludes 7 participants (1 single, 6 pairs) < 2 years

^#^Paired samples t-test, reported as mean difference, *p*-value, Wilcoxon signed-rank test, reported as Z-statistic, *p*-value

#### Individual indicator scores

Table 3 reports the comparison of individual indicator scores derived from the two methods. Healthy fats was the only indicator score to be estimated significantly higher by the MRSDAT overall, and this was consistent across sub-groups. While differences in estimated meat intakes for children did not flow through to differences in meat indicator scores, the SSB indicator was estimated to be 1.53/5 points lower by the MRSDAT in children (*p* = 0.04), with no difference in adults (*p* = 1.00). Fruit indicator scores were almost identical between the methods for children, but the differences in estimated intake did carry through to significantly higher MRSDAT estimated scores for adults (3.29/10 points, *p* = 0.03). There were no significant differences between the other estimates of individual indicator scores.

#### Percentage agreement between methods

Ranking comparisons of the 62 participants with ≥ 2 × 24-h recalls demonstrated moderate agreement (*k* = 0.44, *p* < 0.001), with 58% placed into the same tertile of DGI/DGI-CA score using the different methods. A further 36% were placed into the same tertile ± 1, and only 4 participants (7%) were grossly misclassified. For the whole sample including participants with only single recalls, the agreement was fair (*n* = 98, *k* = 0.38, *p* < 0.001), with 54% placed into the same tertile, 37% into the same tertile ± 1, and 9 participants (9%) grossly misclassified.

## Discussion

This study demonstrates that the updates made to the MRSDAT have improved the agreement between dietary quality index scores derived from it and repeat 24-h recalls [27]. Therefore the DGI-CA/DGI scores estimated from the MRSDAT are a robust measure of diet quality in both children and adults in remote Aboriginal and Torres Strait Islander populations from diverse regions across Australia. Similarly, the food group intakes estimated by the MRSDAT were comparable to those estimated by repeat 24-h recalls, and therefore are again robust measures of intake.

Differences in the overall food group estimates between the methods were primarily seen for SSBs and discretionary foods, with the MRSDAT estimating slightly higher, although not statistically significantly higher, intakes. This is potentially due to these foods being eaten in high volumes when consumed, but not necessarily consumed every day. As such, consumption may not have been captured by the two 24-h recall sessions, while the MRSDAT asks participants to consider their intake over a fortnightly period. For example, if a participant were consuming a full serve of SSB twice a week, this would be captured in the MRSDAT, however unless the 24-h recalls fell on these particular days, this intake would have been missed. This interpretation is supported by higher SSB mean intakes reported for participants with ≥ 2 × 24-h recalls. This same interpretation may also explain the only significant discrepancy in adult food group estimates (fruit intakes) given the reported intakes were so small and infrequent. The adjustments to increase the MRSDAT response options to include smaller portions of meat for children after the previous validation have been successful in correcting the overestimation of meat intakes previously seen for young children [27], with this analysis suggesting MRSDAT underestimates meat intakes by about 0.5 serves. However, this did not translate to discrepancy in DGI-CA scores as the majority of children were meeting recommended intakes regardless of which method was being used to estimate intake.

The demonstrated agreement between the MRSDAT and 24-h recall estimated intakes and dietary quality index scores are comparable, and mostly substantially superior, to other Australian FFQs validated in a variety of populations [28, 46–53]. In some cases, this is difficult to assess however, due to the different methods used to assess validity. Specifically, many studies report Spearman’s and Pearson’s correlation coefficients, which are not appropriate for use in testing agreement [41]. The MRSDAT also substantially outperformed the only other FFQ tested specifically with Aboriginal and Torres Strait Islander children [54]. As a sensitivity analysis, we completed the primary analysis with the outlier participant included, and the Lin’s CCC results were almost identical (Rc 0.53, 95% CI 0.38, 0.66, *n* = 99), and the mean difference in DGI/DGI-CA estimates between the tools was only fractionally higher (0.47/100 points compared to 0.18/100 points, *p* = 1.00, *n* = 99). This provides confidence in the robustness of the results.

The MRSDAT has a number of practical advantages compared to multiple 24-h recalls, and therefore, given the high agreement in estimated intakes and diet quality index scores, could be the preferred choice for future research with remote Aboriginal and Torres Strait Islander peoples. First, experience in the use of MRSDAT with community researchers indicates that with training the MRSDAT could be used by those without specialist training in dietary assessment. Second, the MRSDAT only needs to be completed once, while 24-h recalls should be completed on at least two separate occasions to be considered a reliable measure of usual intake [2]. Third, other work supports the large variability in day-to-day intake in this setting [55] as well as the occurrence of days where little or no food is consumed [9, 13, 16, 27, 56]. Therefore, measuring usual intake over a fortnightly period with the MRSDAT is likely to be more reliable than 24 h recalls in this setting, even when multiple recalls are logistically possible. These three advantages together make the MRSDAT an ideal solution for the reported challenges experienced in collecting dietary data in the Australian National Aboriginal and Torres Strait Islander Nutrition and Physical Activity Survey. The survey used only a single 24-h recall to estimate usual intakes due to challenges around collecting a second dietary recall for this population [57]. Therefore using a method like the MRSDAT would provide a more reliable measure of usual intake, as well as support employment and nutrition capacity building of local community researchers who could be trained to collect data locally. A further advantage of the MRSDAT is the automated scoring enabling immediate intake and diet quality results for participants and researchers. For researchers, the automatic generation of food group intakes and DGI/DGI-CA scores supports vastly more efficient and accurate data analysis; time for data analysis and human error in calculating intakes are both dramatically reduced. For participants the feedback provides a benefit of participating in the research, which our results suggest approximately three-quarters of participants would value. This also potentially extends the MRSDATs applicability into the nutrition practice setting, enabling non-nutrition specialist health staff with appropriate training in its use to provide simple dietary feedback to clients to improve diets. This is especially relevant in remote communities where recruiting and retaining staff, and as such the provision of preventative health services, is challenging [58, 59].

Strengths of this validation study include the diverse geographical footprint of communities included, leading to confidence in the application of the MRSDAT across large areas of Australia. The inclusion of 13 ADG age and life-stage groups again provides confidence in the use of the tool with many different population subgroups, from infants to older adults. While completing a validation study with a ‘gold standard’ known to be biased is a limitation [60], 24-h recalls provide the only realistically achievable alternative method of dietary data collection for a sample of this size. Further, given the above-mentioned challenges in collecting multiple 24-h recalls in this setting, not all participants were able to complete multiple recalls per protocol and therefore the 24-h recall data may not fully capture the same ‘usual’ intake as reported in the MRSDAT. However, the statistical methods used here represent the gold standard for testing agreement [41].

## Conclusion

Updates to the MRSDAT have resulted in estimated food group intake and dietary quality index scores highly comparable to those derived from multiple 24-h recalls. This makes the MRSDAT a robust tool for measuring usual dietary intake with remote Aboriginal and Torres Strait Islander peoples from a diversity of age groups and life stages, and geographical regions of Australia. The MRSDAT has the practical advantages of speed and convenience for research purposes, and the built-in instant dietary feedback feature has application to service delivery settings given the robust results demonstrated in this validation study.

## Data Availability

Restrictions apply to the availability of the data that support the findings of this study which were used under ethics clearance for the current study, and so are not publicly available. Data are however available from the corresponding author upon reasonable request and with permission of all involved parties and with ethics clearance.
